# Discovering Homogeneous Groups from Geo-Tagged Videos

**DOI:** 10.3390/s23094443

**Published:** 2023-05-01

**Authors:** Xuejing Di, Dong June Lew, Kwang Woo Nam

**Affiliations:** School of Computer Science and Engineering, Kunsan National University, 558 Daehak-ro, Gunsan 54150, Republic of Koreardj1301582@kunsan.ac.kr (D.J.L.)

**Keywords:** geo-tagged videos, spatio–temporal databases, clustering, trajectory pattern mining

## Abstract

The popularity of intelligent devices with GPS and digital compasses has generated plentiful videos and images with text tags, timestamps, and geo-references. These digital footprints of travelers record their time and spatial movements and have become indispensable information resources, vital in applications such as how groups of videographers behave and in future-movement prediction. In this paper, first we propose algorithms to discover homogeneous groups from geo-tagged videos with view directions. Second, we extend the density clustering algorithm to support fields-of-view (FoVs) in the geo-tagged videos and propose an optimization model based on a two-level grid-based index. We show the efficiency and effectiveness of the proposed homogeneous-pattern-discovery approach through experimental evaluation on real and synthetic datasets.

## 1. Introduction

With the development and widespread use of localization technologies, the scale and scope of trajectory collection has increased to an unprecedented level [1]. For example, Facebook, Twitter, animal trackers, and vehicles with embedded GPS generate a large amount of geo-tagged information, and large-scale geo-tagged-data analysis is beneficial to applications and services. These technologies enable collection of geo-tagged images/videos from various sources such as eye-tracking data [2,3]. In a previous study, the authors collected eye-tracking data from 1003 images of 15 viewers, recorded each observer’s look tracking path and look position, and used that as ground truth data for training a saliency model using machine learning. The term geo-tagged video refers to a video obtained using a camera device with an embedded GPS sensor to capture the video’s shooting time, location, and direction [4,5,6]. GeoUGV [4] is an extensive geo-tagged video dataset. Each video file in the dataset is accompanied by a geo-tagged metadata sequence, collected by 289 users in more than 20 cities worldwide over a period of 10 years, totaling 2397 videos. Users enable smartphones, portable cameras, or dash cams to capture videos with spatial and time information. Facebook and Twitter can obtain the photographer’s time, location, and scene. A driving recorder can record the scene of a traffic accident. This generated many geo-tagged videos and pictures to help us analyze the time, place, and scene, help us analyze the relationship between the photographers, and predict the things and routes that the photographer is interested in. For example, The Lifelog [7] is a personal record of individual daily life in varying detail for various purposes. The record contains a comprehensive dataset of a human’s activities, and the data could be used to increase knowledge about how people live their lives. In addition, the information recorded in these logs provides favorable conditions for analyzing a user’s interest in scenes and things, and even their gender and preferences.

**Figure 1:**
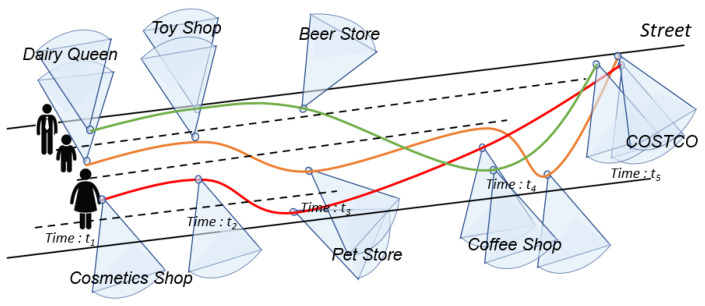
An example of geo-tagged videos with FoVs.

This paper studies the problem of discovering homogeneous groups in geo-tagged videos. For example, when we get a video, we do not know the relationship between the cameramen. Maybe they are family, friends, or strangers. However, we can infer by analyzing these data. Again, this information helps to analyze famous places and who has been to the same places or taken similar routes. For example, Figure 1 shows three different field-of-view (FoV) sequences generated when a family walked on the street. When the family walks on the street, there are many shops around. The father likes to drink beer, so the attention of father focuses on the beer store. The mother likes cosmetics, so the mother focuses her attention on the cosmetics store when passing by a cosmetics store. Finally, a child is more interested in toys or ice cream, so when passing DQ (Dairy Queen), the vision of this child is easily placed in the ice cream shop. Suppose that their eyes stay on the things they are interested in for 5 s. Then, these fields of view form the FoV sequence. In this example, although the distance of family members in space meets a certain threshold, it is assumed that they also meet certain constraints in time. Nevertheless, different FoV directions produced three FoV scenes in different directions. In Figure 1, the father and child look at the same store at $t_{1}$ and $t_{2}$. The child looks at the same store as the mother at $t_{3}$. Furthermore, at the final timestamp direction of the FoV is the same for all three people. In this instance, we assume that they are all less than two meters apart in spatial distance and satisfy certain time constraints. These three geo-tagged videos enable us to regard the people as a group. In summary, our contributions are as follows:

- We propose an algorithm to discover homogeneous groups from geo-tagged videos.
- We propose a density clustering method for geo-tagged FoV clustering based on the DBSCAN method (FDBC).
- We propose an efficient filtering algorithm to reduce the candidates using a two-level grid index (FCBG).
- We prove the efficiency of the proposed algorithm constructs using a real-world shopping dataset [8] and a synthetic dataset which was generated from BDD100K [9].

The rest of our paper is organized as follows. Section 2 summarizes the work related to geo-tagged videos. Section 3 describes preliminary definitions for geo-tagged videos. Section 4 introduces our novel algorithms. Section 5 describes the experimental design and presents experimental observations and results. Finally, we conclude the paper.

## 2. Related Work

Related work can be classified as (i) travel-pattern mining, (ii) geo-tagged videos, (iii) research on clustering of moving objects.

### 2.1. Travel-Pattern Mining

The purpose of travel patterns is to find groups of objects that move together over a period of time, such as flocks [10,11,12], convoys [13,14], swarms [15,16], moving clustering [17], and platoons [18]. Flock and Convoy algorithms differ from other clustering methods because Flock and Convoy have stringent requirements for continuity in time. However, the Convoy algorithm based on density clustering (DBSCAN) breaks the limitation of disks in the Flock algorithm. Unlike Flock and Convoy, the Swarm algorithm is more relaxed about the continuity of time. The Platoon [18] algorithm requires consecutive partial times. In other words, two objects only move together at the beginning and end, and there is no shared moving route for a long period in the middle. For example, two cars only meet while staying at the same gas station, leading to invalid results. The constraints of the moving cluster algorithm [17] require a minimum percentage for co-moving objects between two consecutive timestamps. In order to solve these problems, a unified definition of the common movement patterns has been proposed [19,20,21,22]. Although these methods solve the problem of time and space, these co-movement patterns are all aimed at trajectory analyses. In other words, their purpose is to process only points in each partition, whereas the objects of this paper are geo-tagged videos. In the partition, the processing object is the FoVs polygon. We need to consider the constraints of the co-movement and the viewing area, viewing angle, and viewing direction of FoVs.

### 2.2. Geo-Tagged Videos

Some studies have tried to research effective indexing and search algorithms for FOV scenes. Grid-based indexing based on FOV scenes considered a limited radius and direction to support a *k*-NVS query [23]. The query was proposed to retrieve a recent video segment. MBTR (minimum-boundary-tilt rectangles) in leaf nodes effectively represent the motion scene in the index [24]. A novel geospatial image and video filtering tool (GIFT) was proposed in [25]. Ay et al. [26] proposed a new method of video query. In spatial databases, some studies [27] focus on modeling and representing the visual space of the scene. Kim et al. [28] proposed a representation model for geo-tagged videos with FoV as a vector model. However, none of the above methods enable to solve the problem of homogeneous groups.

### 2.3. Clustering Algorithms

Many clustering algorithms have been proposed for spatial data. K-means clustering first randomly selects K objects as the initial clustering centers, then calculates the distance between each object and each seed-cluster center and assigns each object to the cluster center closest to it [29]. The BIRCH algorithm uses a tree structure to help us cluster quickly [30]. A density-based algorithm contains two parameters (eps and minPts) to identify dense regions based on density reachability [31,32]. Density clustering algorithms are suitable for clusters of arbitrary shapes in geo-tagged photos and videos with FoVs [4,33,34,35]. In order to better avoid the lossy problem, the FoVs clustering method in our paper is based on DBSCAN.

Many density clustering algorithms use grid indexes to reduce the candidates [36,37]. The geographic space is divided into cells of the same size, each cell corresponds to a piece of storage space, and the index item registers the spatial objects that fall into the grid. For example, to query which spatial objects are in a particular spatial range, the grid of the cells is very quickly determined according to the spatial range search for the spatial object in the corresponding cell, and, finally, the spatial object obtained. Ma et al. [23] proposed a novel three-level grid-based index structure and introduced many related query types, including typical spatial queries and some based on a bounded radius and viewing-direction restriction.

## 3. Preliminaries

In this paper, a geo-tagged video is recorded using a camera with a GPS sensor and a magnetic compass. Let VDB = $\left\{v_{1},v_{2},\ldots ,v_{e}\right\}$ be a set of the geo-tagged videos and each video can be described by an id, a set of time, and an FoV sequence v=(id,T,F). Let $T=(t_{1},t_{2},\ldots ,t_{e})$ be a sequence of all timestamps in a geo-video. Additionally, we defined *d* as the intervals of the partition for the timeline. *T* = $\left(t_{1},t_{2},\cdots ,t_{n}\right)$ is a sequence of timestamps after partition, where (T⊂T, $t_{1}\leq t_{n}\leq t_{e}$), i.e.,

(1) T={t|∀t∈T}.

**Definition** **1** (FoV Scene)**.** *Let f denote an FoV scene at time t. The FoV scene is formally defined as:*

(2) f=(id,p,γ,θ,R,t).

**Figure 2:**
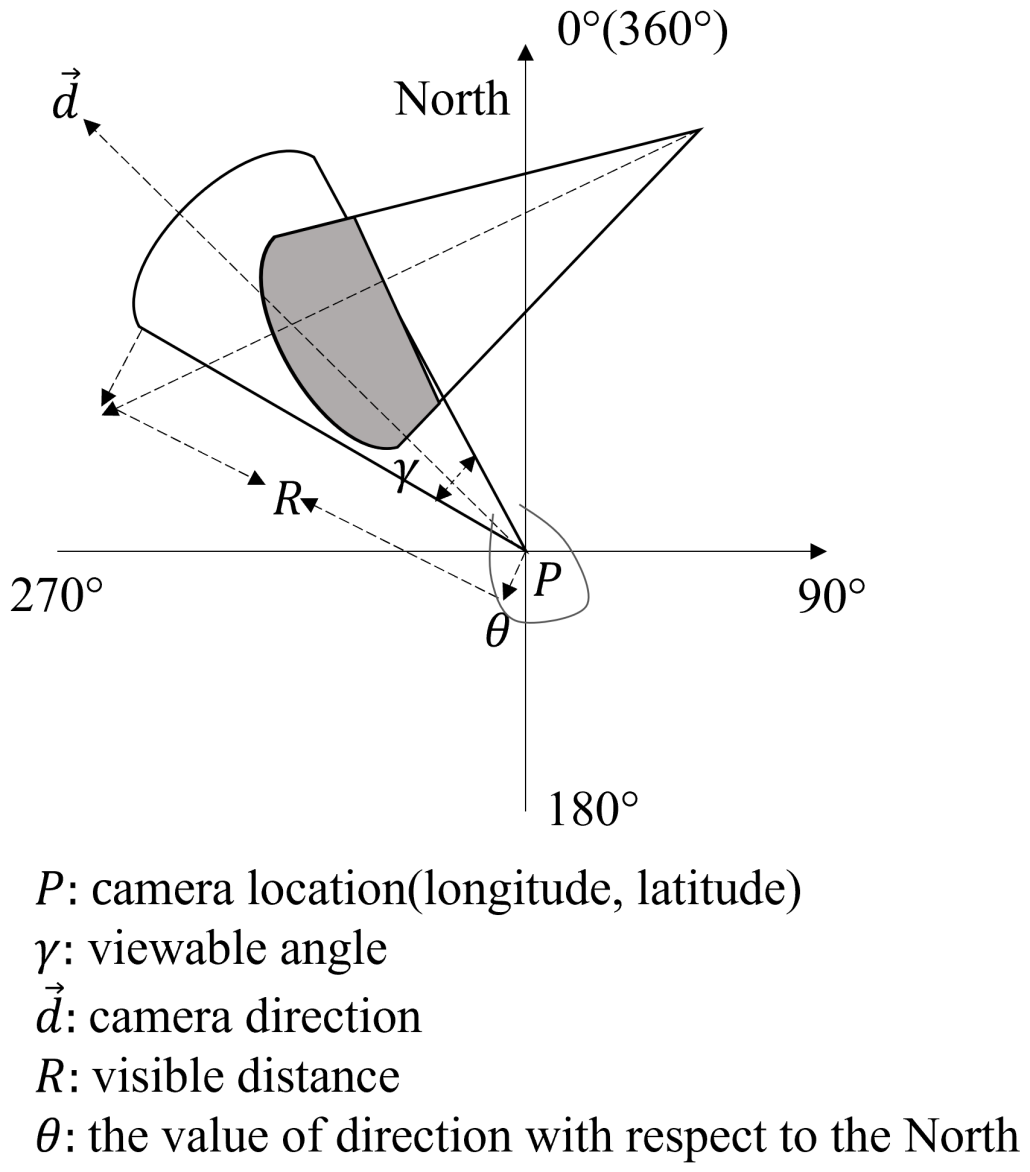
Representative diagram of an FoVScene model in 2 dimensions.

Here, *p* describes the location of camera which consists of longitude and latitude coordinates read from the GPS sensor. γ represents the viewing angle of the camera, θ represents the view-direction value concerning the North, the visible distance is *R* as the maximum distance, and *t* is the timestamp in *T* [27]. The FOV scene and parameters in 2D are shown in Figure 2.

**Definition** **2** (FoV Sequence)**.** *Let F = $\{\left(f_{1}\right)$, $\left(f_{2}\right)$,…, $\left(f_{n})\right\}$ be an FoV sequence in a geo-video, where f is an FoV scence ($t_{n}\in T$). $f_{n}$ is the n-th FoV in a video. The FoV sequence is formally defined as:*

(3) $$F=\{\;f_{v_{e}}\;|\;\forall f_{n}\;\in \;v_{e}\}.$$

**Figure 3:**
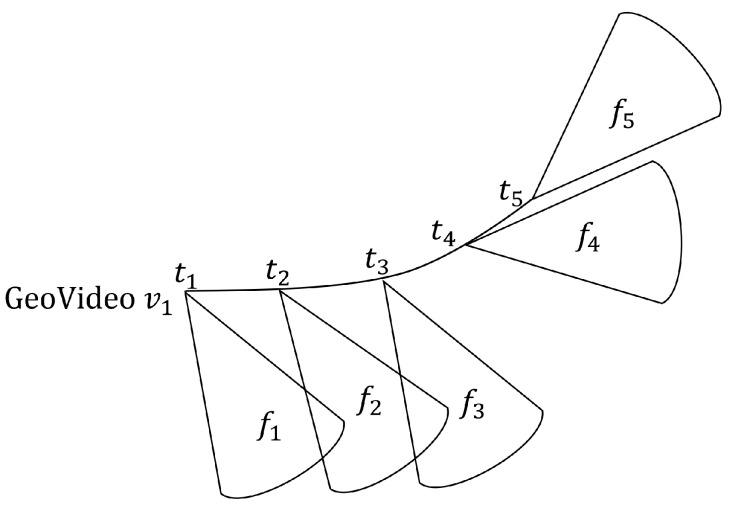
An example of an FoV sequence.

An example of an FoV sequence is shown in Figure 3. In Figure 3, an FoV sequence represents a geo-video $v_{1}$. It consists of each FoV scene and its corresponding timestamp, i.e., *F* = $\{f_{1}$, $f_{2}$, $f_{3}$, $f_{4}$, $f_{5}\}$.

**Definition** **3** (Overlap Coefficient)**.** *The Overlap Coefficient (δ) is the threshold of the common region for two FoVs. Given two FoVs of two videos (f and $f^{\prime }$), the common area coefficient between f and $f^{\prime }$ is defined as follows:*

(4) $$Area_{inter}\left(f,f^{\prime }\right)=\frac{\left|f\cap f^{\prime }\right|}{\left|f\right|+\left|f^{\prime }\right|-\left|f\cap f^{\prime }\right|}$$

*where δ∈ (0, 1], |f| and $|f^{\prime }|$ are the areas of two FoVs, and $|f\cap f^{\prime }|.$*

Formula (4) is defined based on the Jaccard index [38]. An example is shown in Figure 2; the black region represents the intersection of the area between two FoVs (*f* and $f^{\prime }$ in the same snapshot). If the proportion of overlapping area ($Area_{inter}\left(f,f^{\prime }\right)$ is more significant than or equal to the threshold δ, the two FoVs satisfy the intersection-of-area constraint.

**Definition** **4** (Closeness)**.** *Given two FoVs f and $f^{\prime }$ in a snapshot $s_{t}$. Closeness is if the distance between two FoVs is less than the maximum distance threshold ϵ; moreover, it is satisfied if the intersection of the viewable area of the two FoVs is greater than the minimum area intersection threshold $Area_{inter}$, as shown in Formula (4). The closeness of FoVs is defined as*

(5) $$\{f|\exists f^{\prime }\in s_{t},Area_{inter}\left(f,f^{\prime }\right)\geq \delta \wedge dist\left(f_{p},{f\prime }_{p}\right)\leq \varepsilon \},$$

*where $dist(f_{p},f_{p}^{\prime })$ denotes the minimal distance between the positions of f and $f^{\prime }$.*

**Definition** **5** (Clusters of FoVs)**.** *Let SC = {$SC_{1}$, $SC_{2}$,…, $SC_{t}$} be the collection of FoVs from snapshots at the timestamp in $\left\{t_{1},t_{2},\cdots ,t_{n}\right\}$, where $SC_{t}=\left\{C_{1},C_{2},\cdots ,C_{n}\right\}$ denotes a set of clusters in a snapshot $S_{t}$, where $C_{n}$ is an FoV cluster. If the number of FoVs in $SC_{t}$ that are satisfying Formula (5) is at least $min_{o}$, then these FoVs will form a cluster, where $min_{o}$ is the minimal numbers of FoVs.*

**Figure 4:**
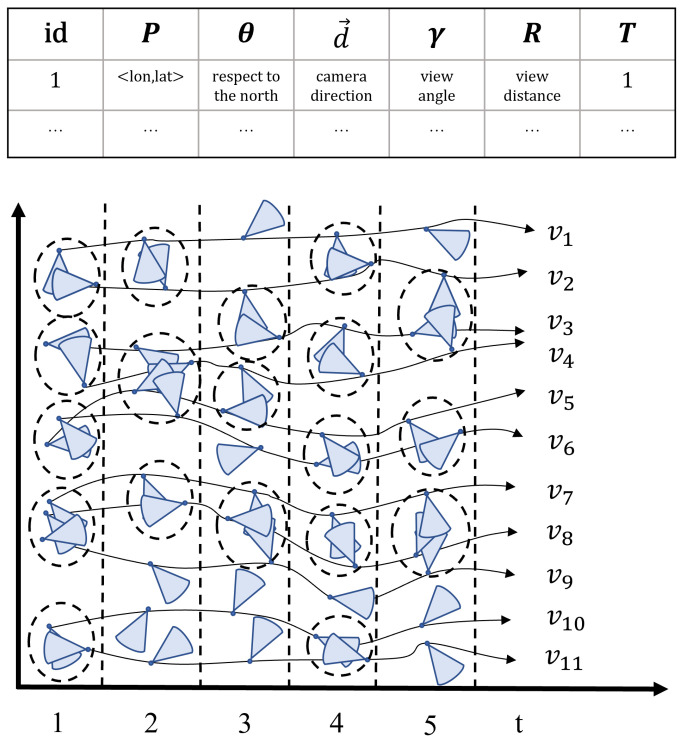
Examples of snapshots in various FoV videos.

For example, the FoVs satisfy the distance threshold as Figure 4 shows. If $min_{o}$ = 2, we can obtain five clusters $\{(f_{v1}$, $f_{v2})$, $((f_{v3}$, $f_{v4})$, $(f_{v5}$, $f_{v6})$, $(f_{v7}$, $f_{v8}$, $f_{v9})$, $(f_{v10}$, $f_{v11})\}$ in the snapshot at $t_{1}$. However, we only can capture one cluster if we change to $min_{o}$ = 3, i.e., $(f_{v7}$, $f_{v8}$, $f_{v9})$.

Let the time segment $T_{i}$ be the *i*-th time segment of *T*, i.e., ($T_{i}\in T$). If ∃T[n] that satisfies $\left(T\left[n+1\right]=T\left[n\right]+1\right)$, (1≤i≤n), then $T_{n}$ is the FoV sequential segment, where n is the timestamp of the *n*-th snapshot. When *T* is composed of *s* sequential time segments, *T* is *s*-succession. Further, if every adjacent FoV’s time, such as ($T\left[n\right]$, T[n+1]∈T), to satisfy the difference value is no bigger than g, i.e., ($1\leq n<\left|T\right|$, $T\left[n+1\right]$−T[n]≤g), then *T* is g-connection. Additionally, the groups also need another constraint *k*; it is the minimal number of FoVs belonging to the same cluster.

For example, given two times segments $T_{1}$ = (5, 6, 7), $T_{2}$ = (5, 6, 8), and *T* = $T_{1}$, $T_{2}$, $T_{1}$ is an FoV sequential segment, but $T_{2}$ is not because the 7 is lost in $T_{2}$. In the above demonstrate, given a segment *T* = (5, 6, 8, 9, 10), Segment *T* is two-sequential and two-connection. The minimal length segment (5, 6) of T is 2, therefore, the two-sequential FoV segments are $T_{1}$ = (5, 6) and $T_{2}$ = (8, 9, 10) in *T*.

**Definition** **6** (Homogeneous Groups)**.** *Let P = $\{P_{1}$, $P_{2}$,…, $P_{n}\}$ be the homogeneous groups of geo-videos required to satisfy five constraints as follows:*

 *(1)* ***FC (FoVs Cluster):*** *the FoVs in a set of geo-tagged videos belong to the same cluster at each timestamp of T.*
 *(2)* ***Occurrence:*** *k is the minimal number of occurrence for FoVs belong to the same cluster.*
 *(3)* ***Significance:*** *|C| ≥ $min_{o}$.*
 *(4)* **Succession:** *T is s-sequential.*
 *(5)* **Connection:** *T is g-connected.*

Consider the example in Figure 4; a dotted circle denotes a cluster. Given $min_{o}$ = 2, *k* = 3, *s* = 2, *g* = 2 and $area_{inter}$ = 0.5, then we are able to capture three homogeneous groups P = $\left\{\left\{v_{3},v_{4}\right\},\left\{v_{5},v_{6}\right\},\left\{v_{7},v_{8}\right\}\right\}$ for *T* = <$t_{1}$, $t_{2}$, $t_{4}$, $t_{5}$>. In other words, $\left\{v_{3},v_{4}\right\}$, $\left\{v_{5},v_{6}\right\}$, and $\left\{v_{7},v_{8}\right\}$ belong to the same cluster at timestamps 1, 2, 4, and 5.

**Definition** **7** (Grid)**.** *Let key be the ID of the global grid cells. Let Gr = $\{g_{1}$, $g_{2}$,…, $g_{n}\}$ be an integer grid. Then ($min_{x},min_{y},max_{x},max_{y}$) are the boundaries in a snapshot $S_{t}$ (x represents longitude, y represents latitude). For each empty cell, g = (key, false, f). Then, let e be an edge of one FoV, Seg be the set of segments of an edge, SID be a set of cells’ ID of segments in an edge, and Keys be the cells’ ID covered by f. Gr is the index set of the finally obtained FoVs. For each key in Keys, if FoV f be inserted into the cell g, g = (key, true, f) is the grid cell of Gr.*

**Definition** **8** (Grid-clustering)**.** *Let SC = $\{SC_{1}$, $SC_{2}$,…, $SC_{t}\}$ be the clusters of FoVs in snapshots $S_{t}$ based on the grid index. Let $f_{MBR}$ be the minimum-bounding rectangle (MBR) of the FoV f. We use $V_{r}$ to represent a collection of all grid cell IDs covered by MBR, such that*

(6) $$RangeC\left(f_{MBR},V_{r}\right)=\left\{f|\forall f_{id}\in V_{r},\right\}$$

*where the RangeC are candidates of Grid-clustering.*

The Table 1 summarizes the notations used in the paper.

## 4. Proposed Approach

### 4.1. Naive Algorithm Design

The skeleton of the homogeneous-group mining in the geographic video database VDB contains three stages. Firstly, we transform the geo-tagged video into snapshots, i.e., a snapshot at timestamp 1, a snapshot at timestamp 2,…, a snapshot at timestamp *n*. Secondly, we cluster the FoVs for each snapshot at each timestamp based on density clustering (FDBC). Due to limited space, we omit the algorithm in this paper.

In Algorithm 1, FoVs are arbitrarily selected from each snapshot until all FoVs have been visited. If there are at least $min_{o}$ FoVs within a radius of ϵ, i.e., ($F=\{f^{\prime }|dist\left(f,f^{\prime }\in S_{t}\right)\leq \epsilon \}\wedge |F|\geq min_{o}$), these FoVs are considered as part of the candidate cluster, and ExpandFoVCluster generates a more refined cluster that satisfies the constraint. Next, the overlap between FoVs in the cluster is determined by the overlapArea function. Then, if the number of FoVs that satisfy the intersecting constraint is greater than the minimum FoV number $min_{o}$, they are inserted into $SC_{temp}$ as candidates. Otherwise, they are marked as noise.
**Algorithm** **1:** FDBC($S_{t}$, ϵ, $min_{o}$, δ).
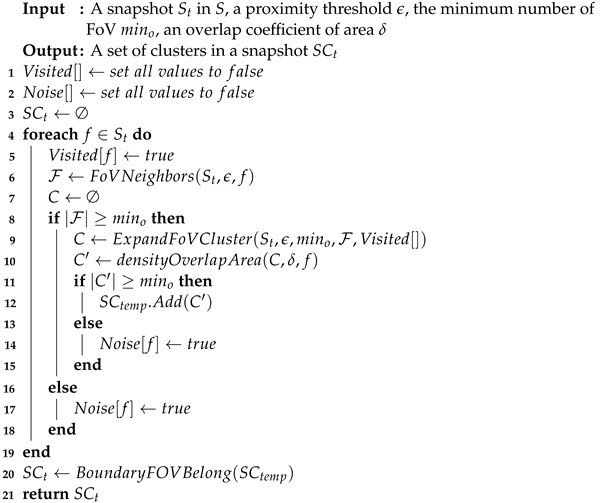


The detail for overlapArea is described as Definition 3, detecting whether a common area exists between two FoVs. We calculate the overlap area coefficient (δ) after judging the distance between two FoVs. However, it is not obligatory that as long as two FoVs intersect they view the same thing. In consequence of this, we follow to the intersection area $Area_{inter}$. A result of common-area weight of no less than $Area_{inter}$($\delta \leq Area_{inter}$) is stored in the list *C* that is used for ExpandFoVCluster. The ExpandFoVCluster is depicted in Algorithm 2.
**Algorithm** **2:** ExpandFoVCluster($S_{t}$, ϵ, $min_{o}$, F, Visited[]).
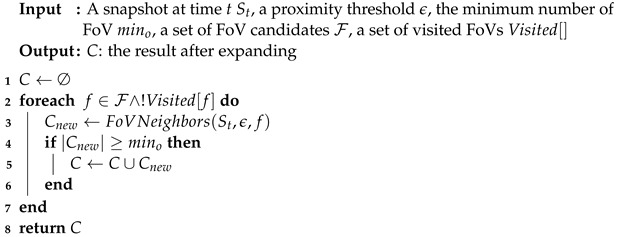


Algorithm 2 describes the expansion of the FoV cluster. For each FoV in *C* that has not been visited, mark it as visited and look for its neighbor $C_{new}$. If the number in $C_{new}$ is not less than the minimum FoV number, use it as a candidate set for overlapping area calculation, and calculate the FoV afterward. If the number meets $min_{o}$, it will be merged with the previously generated cluster, and the operation will be repeated until reaching the boundary of the cluster. ExpandFoVCluster is the working core of the algorithm and an essential operation is used for spatial query, retrieving all the FoVs belonging to a given region of the space and having a common viewable region. It can be shown that clusters are invariant with respect to the order of FoV selection in ExpandFoVCluster, and that the complexity of FDBC is $O(N^{2})$ the worst, where *N* is the number of FoVs.

Finally, we discovered groups from videos according to clusters of snapshot’s timestamps. It is described in Algorithm 3. Before that, we introduced that the conditions for grouping geo-tagged videos are view field, location, and the time the people move together. In this phase, we use constraints to judge these geo-tagged videos; whether they are a group. If geo-tagged video objects in the same cluster meet the time constraints, we regard them as a group.

In more detail, the time cost of the FDBC algorithm can be expressed as follows:

(7) $$Cost_{FDBC}=\Theta \left(S_{t},\epsilon \right)+\Pi \left(S_{t},\epsilon ,min_{o}\right)+\Gamma \left(C,\delta \right)+\Lambda \left(SC\right),$$

where $\Theta (S_{t},\epsilon )$ is the time cost of FovNeighbors, $\Pi (S_{t},\epsilon ,min_{o})$ is the time cost of ExpandFoVCluster, Γ(C,δ) is the time cost of densityoverlapArea, and Λ(SC) is the time cost of BoundaryFOVBelong.

The $\Theta (S_{t},\epsilon )$ representation is the time it takes to calculate the distance between each field of view (fov) of $S_{t}$ and determine whether the distance is part of the adjacent fov by making sure it is within the radius ϵ. The time required to construct the neighboring fov depends on the size of the set |F| in $S_{t}$ and the radius, as shown in Equation (8). As the radius ϵ increases, the number of candidate fovs increases, resulting in a longer overall algorithm execution time.

(8) $$\Theta \left(S_{t},\epsilon \right)\approx \left|F\right|*\theta_{F,\epsilon }$$

where $\theta_{F,\epsilon }$ represents the time required to calculate the neighbors associated with fov. $\Pi (S_{t},\epsilon ,min_{o})$ means the time cost to ExpandFovCluster satisfiying $min_{o}$ and ϵ from a dataset F by FovNeighbors. The time cost can be estimated approximately using the following equation:

(9) $$\Pi \left(S_{t},\epsilon ,min_{o}\right)\approx |F|*\pi \approx |F|\;*\;\theta_{S_{t},\epsilon }*\pi ,$$

where F is a set of candidates in *F*, and π is the average time taken to perform ExpandFoVCluster. The number of $min_{o}$ affects the overall running time of the algorithm. $\theta_{S_{t},\epsilon }$ is a selectivity rate of $\Theta (S_{t},\epsilon )$.

(10) $$\Gamma \left(C,\delta \right)\propto |F|*\pi_{S_{t},\epsilon ,min_{o}}$$

Γ(C,δ) means the time cost to do densityOverlapArea for *C* with the parameter δ using Equation (4), where $\pi_{S_{t},\epsilon ,min_{o}}$ is a selectivity rate of $\Pi (S_{t},\epsilon ,min_{o})$.

(11) $$\Lambda \left(SC\right)\propto |F|*\pi_{S_{t},\epsilon ,min_{o}}*\gamma_{c,\delta }$$

Λ(SC) means the time cost to do BoundaryFOVBelong. Each operation is performed for each element in the cluster, resulting in a time complexity of $n^{2}$, and the algorithm execution time is influenced by the number of fov in each cluster where $\gamma_{c,\delta }$ is a selectivity rate of Γ(C,δ).
**Algorithm** **3:** BoundaryFoVBelong($SC_{temp}$, $min_{o}$).
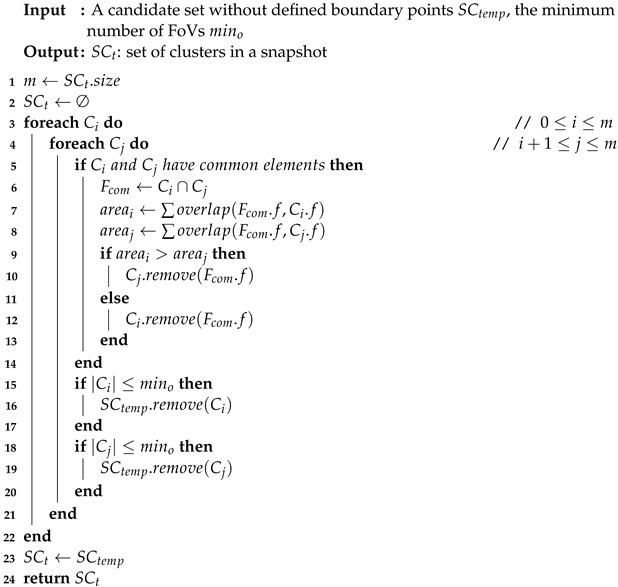


Scan clusters in the order of each snapshot with the snapshots after it, and detect the FoV belonging to the same geo-video in the cluster between snapshots. Add these FoVs into the A.C. When the number of FoVs |(A.C)| in A.C is not greater than $min_{o}$, put the timestamps of FoVs into A.T. It is initialized using the common FoVs in clusters with a size no smaller than $min_{o}$. After that, generate new candidates A by extending. Next, for every group candidate *A* in A, we execute the CandidatesFilter to obtain the final homogeneous groups, which is described in detail in Algorithm 4.

**Definition** **9** (Groups Candidates)**.** *Considering Definitions 5 and 6, the homogeneous groups need to satisfy the Closeness and certain time constraints. Therefore, let A = $\{a_{1}$,…, $a_{m}\}$ be a set of the candidate set, and let $a_{i}$ = <C, T> be a candidate where $a_{i}$ is a sub-candidate of A.*

**Algorithm** **4:** MiningHomogenousGroups(SC, $min_{o}$, *k*, *s*, *g*).

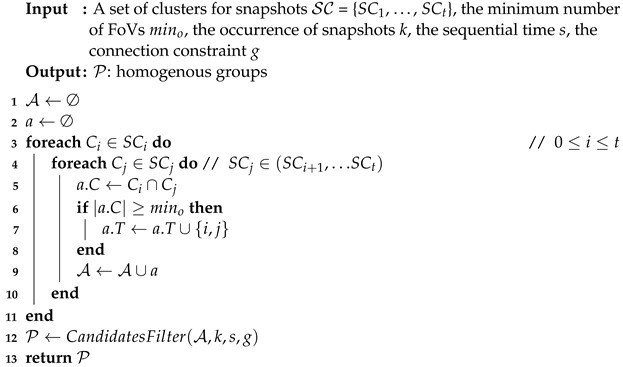



In Algorithm 4, we judge the succession and connection. For succession, the most significant gap between the two time-segments is whether it is lower than *g*. If not, remove it. Furthermore, we remove it if the sequential time is bigger or equal to *s*.

Then, we judge the occurrence that the minimal number of occurrences for FoVs belongs to the same cluster in each segment; if it is less than *k*, we also remove *a* from A. After that, all the homogeneous groups are stored in P. As an example, a glance at Table 2 illustrates an instance. If we set the parameters $min_{o}$ = 2, *s* = 1, *g* = 2, *k* = 2, and δ = 0.5, then all of the groups that we would capture are shown in Table 2.

For the groups part, we assume the number of clusters is *N* and the computational complexity is $N^{2}+\left|T^{2}\right|$.

### 4.2. Performance Enhancement Using a Grid Index Approach

This paper proposes constructing a grid index based on area-density clustering for each snapshot. Because FoV clustering is different from point clustering, an essential condition needs to calculate the overlap area. The purpose of using the grid is to use MBR to narrow the scope of the calculation further. The details are described in the following.

**Figure 5:**
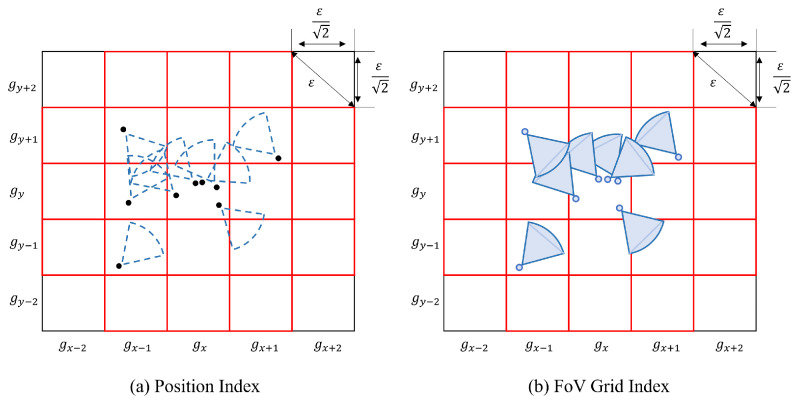
An example of FoV clustering using a grid index.

For example, if we want to find all the FoVs associated with a specific FoV, we need to compute the $f_{MBR}$. It is represented by the area in the red box in Figure 5a. Furthermore, we can get the id of the cell that the FoV to be queried belongs to. The process is depicted in Algorithm 5. After that, we need to judge whether the number of FoVs in $V_{f}$ is greater than the threshold $min_{o}$. If the quantity condition is met, we execute the second level. The second level further divides each grid in the first level into c×c sub-cells; it in depicted in Figure 5b. The width τ of a sub cell is as follows:

(12) $$\tau =(\epsilon /\sqrt{2})/c$$

**Algorithm** **5:** FCBG($S_{t}$, ϵ, $min_{o}$, *c*, δ).
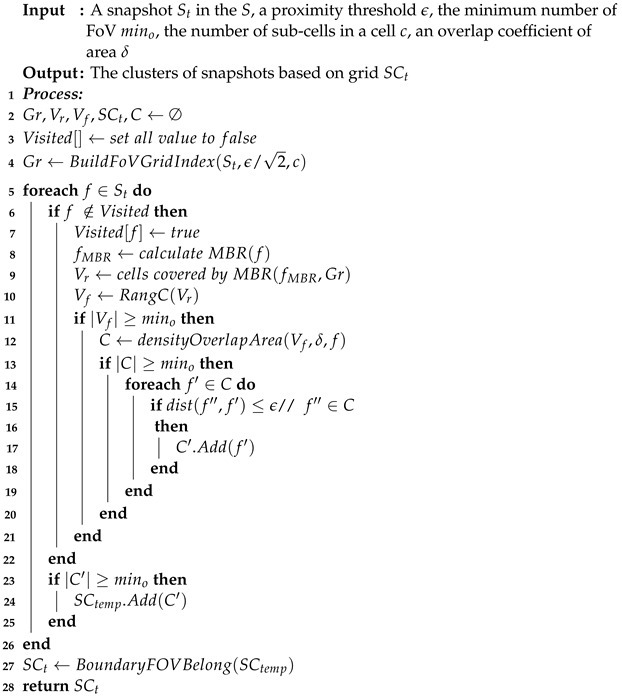


These sub-cells are used to calculate the intersection of the area between two FoVs. These FoVs will be marked as visited if they satisfy the intersection condition. Next, we need to judge the distance again. If the distance between *f* and $f^{\prime }$ is greater than the distance threshold ϵ, remove $f^{\prime }$ from candidate cluster *C*. Finally, if the number of FoVs in *C* is no less than $min_{o}$, we obtain one of the clusters in a snapshot. The computational cost of FCBG is O(NlogN) with a grid index, where *N* is the number FoVs in the snapshot.

In more detail, the time cost of the FDBC algorithm can be expressed as follows:

(13) $$Cost_{FCBG}=\Phi \left(S_{t},\epsilon ,c\right)+\Gamma \left(C,\delta \right)+\Lambda \left(SC\right),$$

where $\Phi (S_{t},\epsilon )$ is the time cost of BuildFovGridIndex and calculates MBR, $\Pi (S_{t},\epsilon ,min_{o})$ is the time cost of ExpandFoVCluster, and Γ(C,δ) is the time cost of densityoverlapArea, and Λ(SC) is the time cost of BoundaryFOVBelong.

## 5. Experimental Evaluation

We describe the selection of the parameters for clustering and evaluate the FoV clustering’s effectiveness based on the DBSCAN clustering (FDBC) algorithm and the FoV clustering based on Grid (FCBG) by experimenting with two datasets. Moreover, the investigation of the analogous interest groups captured by utilizing two clustering methods is discussed in this chapter. The description of the experimental setting and the geo-tagged-videos datasets are described in Section 5.1. The performance evaluation of clustering under different datasets by attributing various values of the parameters is described in Section 5.2.

### 5.1. Experiment Setting

All experiments were performed using an Intel (R) Core (TM) i7-3770 CPU machine with 16 GB of RAM and 20 TB of hard disk space. The algorithms were implemented based on JDK1.8.

Datasets: This experiment was based on two types of datasets, the BDD dataset [9] and the geo-tagged-videos data constructed based on a real-world shopping dataset for trajectories. The data sample was recorded per second. The experiment set the different intervals of the duration depending on the datasets used in our experiment. The statistics of the datasets are listed in Table 3.

### 5.2. Performance of Clustering

**Selection of $min_{o}$** Value: The rule of thumb [39] is Formula (14), where dim represents the dimension of the data to be clustered.

(14) $$min_{o}\geq dim+1$$

If $min_{o}$ = 1, each independent point is a cluster. On the other hand, if $min_{o}\leq 2$, the result is the same as the hierarchical distance to the nearest neighbor. Therefore, $min_{o}$ must be a value greater than or equal to 3. If the selected value is too small, the result in the sparse cluster will be considered as a boundary point because the density is less than $min_{o}$, and will not be used for further expansion of the class; if the value is too large, two adjacent clusters with higher density may be merged into the same cluster. At the same time, the value of $min_{o}$ should also be selected according to the size of the dataset. In this paper, due to our research it is based on the two-dimensional FoV model, hence, we set $min_{o}$ to 4, 8, 12, 16, and 20.

Selection of ϵ value: If the value of ϵ is too small, most of the data are missed. On the contrary, if the parameter setting is too large, multiple clusters or most objects are merged into the same cluster. In our work, we used the Euclidean method (as following Formula (15) [40] to calculate the *k*-distance graph. That is, given the parameter *k*, calculate the *k*-th nearest neighbor distance corresponding to each position and and sort in descending order; call this picture a sorted *k*-distance graph. Here, *k* = $min_{o}$.

(15) $$dist\left(f_{p},{f^{\prime }}_{p}\right)=\sqrt{{\left(f_{p.x}-f_{p.x}\right)}^{2}+{\left(f_{p.y}-{f^{\prime }}_{p.y}\right)}^{2}}$$

One of the conditions for two FoVs to have a common area is that the distance between the two positions is not greater than ϵ. Therefore, we only discuss distances within 100. The number of distances is in different ranges. This paper utilized a histogram to display the frequency of the distance range from 2.3 to 100 and choose the average in adjacent partitions with significant differences in frequency of distance as the parameter, as per Formula (16), where N is the frequency of distance.

(16) $$\varepsilon =\frac{1}{N}\sum_{j=1}^{N}{\left(\frac{\sum_{i=1}^{k}{dist}_{i}\left(f_{p},{f^{\prime }}_{p}\right)}{k}\right)}_{j}$$

**Figure 6:**
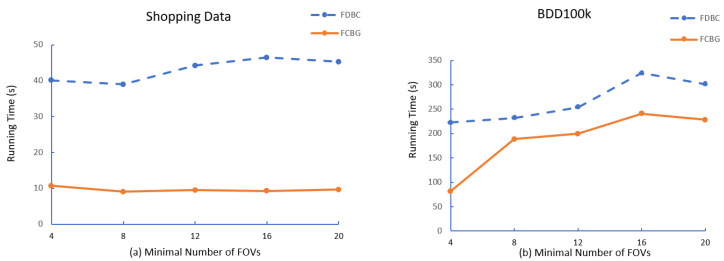
Effect of various $min_{o}$.

**Figure 7:**
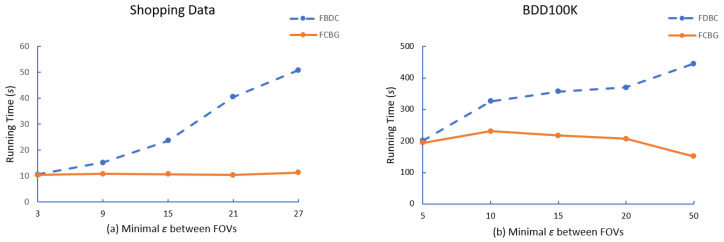
Effect of various ϵ.

**Figure 8:**
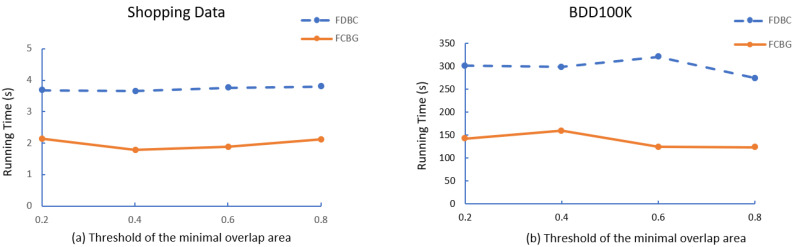
Effect of various δ.

After that, we compare the running time of FDBC and FCBG with different parameters, i.e., the minimal number of FoV ($min_{o}$), minimal eps between FoVs (ϵ), minimal overlap area (δ). As shown in Figure 6, Figure 7 and Figure 8, FCBG is faster than FDBC under the same parameters.

**The effect of $min_{o}$**: To test the influence of the $min_{o}$ threshold, (Shopping data: fixed ϵ = 3, δ = 0.5; BDD100K: ϵ = 50, δ = 0.5) we changed the minimal number of FoV threshold from 4 to 20. Figure 6 shows the clustering cost of the algorithm FDBC and the algorithm FCBG based on the $min_{o}$ threshold. According to Figure 6, we observe that the running time of FDBC becomes longer as the minimal FoV threshold increases because the first step of FDBC is to find the nearest neighbor of ϵ. It calculates FoV one by one and then judges the minimal number which increases the computation. However, FCBG, according to the MBR, reduces the region of calculation. After $min_{o}$ = 12, the time cost rises smoothly in Shopping data because after the FDBC calculates the distance, if $min_{o}$ is not satisfied, the following mark calculation is required. Therefore, the time cost has a slow rise depending on the different distribution densities of the data. It can be seen that there are not many clusters that meet the 12 FoVs as a class. In addition, the BDD100K shows that FCBG also has a significant increase with the increase in $min_{o}$; but overall, the time of FCBG is lower than that of FDBC.

**The effect of ϵ**: To test the influence of the distance threshold, we fixed Shopping data and BDD100K to $min_{o}=4$ and δ = 0.5, and changed the distance threshold from 3 m to 27 m. Figure 7 shows the clustering cost of the algorithm FDBC and the algorithm FCBG based on the distance threshold. According to Figure 7, we observe that the running time of FDBC becomes longer as the distance threshold increases because the first step of FDBC is to find the nearest neighbor of $min_{o}$ within the distance threshold. A more extensive calculation is needed as the distance increases because this means the number of FoVs that need to be calculated will increase. On the contrary, distance is not the primary influencing factor of FCBG. As FCBG is based on the MBR of FoV and based on the grid index, it calculates the overlap coefficient δ = 0.5 and obtains $min_{o}$ FoVs whose distance threshold is not greater than eps. Therefore, the time cost of FCBG does not fluctuate significantly with an increase in ϵ, as shown in the figure. In addition, in terms of running time, the efficiency of our optimized FCBG is better than that of FDBC.

**The effect of δ**: To evaluate the effect of varying δ, we fixed the threshold (Shopping: ϵ = 3 m, $min_{o}$ = 4; BDD100K: ϵ = 50 m, $min_{o}$ = 4), and set δ from 0.2 to 0.4. Figure 8 illustrates the clustering time of the two algorithms. In the experiment, the running time depends on the distribution of the data. When the density that satisfies the overlap threshold is large, the amount of calculation is significant, and vice versa. Nevertheless, the overall efficiency of FCBG is still higher than that of FDBC.

**Figure 9:**
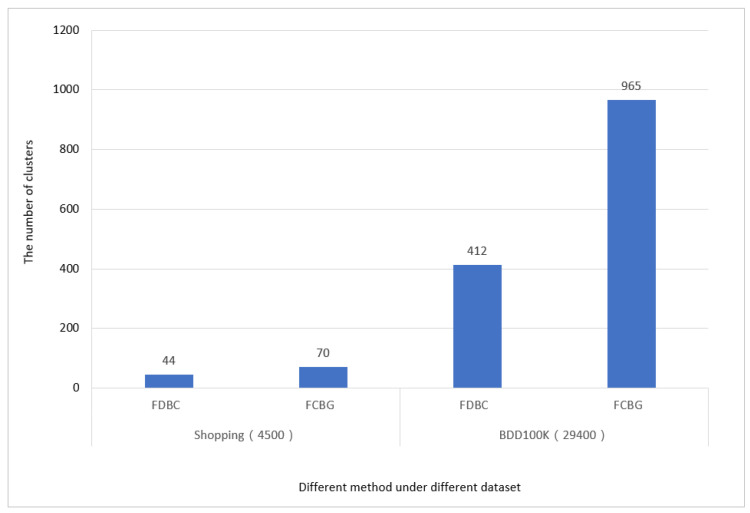
The clustering performance with FDBC and FCBG.

In order to further observe the clustering performance, this experiment studies the cluster size to better compare the clustering effects of different methods on different datasets. That is, the different datasets are used to cluster FoVs for each snapshot in $S=\{S_{1}$, $S_{2}$,…, $S_{t}\}$ at timestamp t. We average the number of clusters generated in $S_{t}$ to compare the clustering effects of FBDC and FCBG, as Figure 9 shows.

In Figure 9, since the number of different shopping videos is less than the number of videos in BDD100K, the obtained shopping cluster size is less than that of BDD100K. According to the description in Figure 5, the number of clusters that the algorithm FCBG can capture is higher than the number captured by the algorithm FDBC in both data sets.

## 6. Conclusions

In this paper, we firstly proposed novel algorithms to discover homogeneous groups from geo-tagged videos, using view directions. Secondly, we extended the density clustering algorithm to support FoV clustering and proposed an optimization model based on a two-level grid-based index structure. For experimental validation, we used a real shopping dataset and an FoV-extended synthetic video dataset. The synthetic video set was extended from the real BDD dataset. The results show that our algorithms are effective to discover homogeneous groups from geo-tagged videos using view directions. It is expected that cameras with directional sensors will become much more popular in the future, and we believe that the new algorithms proposed in this paper can be heavily utilized for social media analysis.

## Figures and Tables

**Table 1:** Frequently used notations.

Notation	Description
F	FoV sequence in a geo-video
T	Sequence of all timestamps after partition in a geo-video
δ	Overlap coefficient of common region for two FoVS
SC	FoV collection of snapshots at the timestamp
C	Clusters in a snapshot $S_{t}$
*k*	Minimal number of occurrence for FoVs belonging to the same cluster
$min_{o}$	Minimal number of FoVs

**Table 2:** The homogeneous groups corresponding to Figure 4.

Groups						
GroupsID	1	2	3	4	5	6
VideoID	1, 2	3, 4	5, 6	7, 8	7, 8, 9	7, 9
SnapshotID	1, 2, 4	1, 2, 4, 5	1, 2, 4, 5	1, 2, 3, 4, 5	1, 3, 5	1, 3, 5

**Table 3:** The Detail of Datasets.

Statistics	Shopping	BDD100K
Number of geo-videos	4500	29,490
The longest time-domain length of video	42,000	30,000
The total of FoVs	135,000	1,238,580
The number of snapshots	10	40
Cluster size	44/70	412/965

## Data Availability

Not applicable.
